# Macrophages as IL-25/IL-33-Responsive Cells Play an Important Role in the Induction of Type 2 Immunity

**DOI:** 10.1371/journal.pone.0059441

**Published:** 2013-03-25

**Authors:** Zhonghan Yang, Viktoriya Grinchuk, Joseph F. Urban, Jennifer Bohl, Rex Sun, Luigi Notari, Shu Yan, Thirumalai Ramalingam, Achsah D. Keegan, Thomas A. Wynn, Terez Shea-Donohue, Aiping Zhao

**Affiliations:** 1 Department of Medicine and the Mucosal Biology Research Center, University of Maryland School of Medicine, Baltimore, Maryland, United States of America; 2 Department of Biochemistry, Zhongshan Medical School, Sun Yat-sen University, Guangzhou, China; 3 U.S. Department of Agriculture, Agricultural Research Service, Beltsville Human Nutrition Research Center, Diet, Genomics, and Immunology Laboratory, Beltsville, Maryland, United States of America; 4 Center for Vascular and Inflammatory Diseases, University of Maryland School of Medicine, Baltimore, Maryland, United States of America; 5 Division of Parasitology, National Institute of Allergy and Infectious Diseases, National Institutes of Health, Bethesda, Maryland, United States of America; McMaster University, Canada

## Abstract

Type 2 immunity is essential for host protection against nematode infection but is detrimental in allergic inflammation or asthma. There is a major research focus on the effector molecules and specific cell types involved in the initiation of type 2 immunity. Recent work has implicated an important role of epithelial-derived cytokines, IL-25 and IL-33, acting on innate immune cells that are believed to be the initial sources of type 2 cytokines IL-4/IL-5/IL-13. The identities of the cell types that mediate the effects of IL-25/IL-33, however, remain to be fully elucidated. In the present study, we demonstrate that macrophages as IL-25/IL-33-responsive cells play an important role in inducing type 2 immunity using both *in vitro* and *in vivo* approaches. Macrophages produced type 2 cytokines IL-5 and IL-13 in response to the stimulation of IL-25/IL-33 *in vitro*, or were the IL-13-producing cells in mice administrated with exogenous IL-33 or infected with *Heligmosomoides bakeri*. In addition, IL-33 induced alternative activation of macrophages primarily through autocrine IL-13 activating the IL-4Rα-STAT6 pathway. Moreover, depletion of macrophages attenuated the IL-25/IL-33-induced type 2 immunity in mice, while adoptive transfer of IL-33-activated macrophages into mice with a chronic *Heligmosomoides bakeri* infection induced worm expulsion accompanied by a potent type 2 protective immune response. Thus, macrophages represent a unique population of the innate immune cells pivotal to type 2 immunity and a potential therapeutic target in controlling type 2 immunity-mediated inflammatory pathologies.

## Introduction

Type 2 immunity plays a crucial role in host defense against nematode infection and pathogenesis of atopic diseases such as allergy and asthma. As opposed to the well-established Th1 immunity, the cellular and molecular events occurring at the early phase of type 2 immunity remain unclear despite intensive research in recent years. Both IL-25 and IL-33 are epithelium-derived cytokines that are important in initiating type 2 immunity through the induction of downstream IL-4/IL-5/IL-13 [1], [2], however, the specific cells that respond to these cytokines have not been fully identified. Early studies suggested that these IL-25-responsive cells were an unidentified population of IL-4/5/13-producing non-B non-T cells [3]. Several novel innate cell populations including “natural helper cells”, “nuocytes”, and “innate helper type 2 cells”, collectively referred as type 2 innate lymphoid cells (ILC2), were shown to respond to IL-25/IL-33 and produce type 2 cytokines [4]–[7]. It is noteworthy that most of these cell types are observed rarely in tissues under steady-state conditions.

Macrophages are major innate immune cells distributed in almost all tissues/organs throughout the body. One of the largest population of macrophages resides in the gastrointestinal tract [8], where they are located strategically beneath the intestinal epithelium and play a central role in gut homeostasis and host defense against various pathogens [9]. While it is well established that macrophages are one of the major producers for Th1-associated cytokines/mediators implicated in variety of inflammatory diseases including inflammatory bowel disease [10], [11], the role of macrophages in type 2 immunity remains poorly understood. Previous studies show that alternatively activated macrophages (M2) are required for host protective immunity against nematode infection [12], [13]. In response to a Th2-dominant enteric nematode infection, macrophages are recruited to the site of infection and become M2 that play a crucial role in the intestinal smooth muscle hypercontractility/hypertrophy and perhaps in the resolution of inflammation [12], [13]. Several previous *in vivo* and *in vitro* studies showed that macrophages produce IL-4 and IL-13 after viral infection or when co-cultured with NKT cells [14]–[17]. A very recent paper further demonstrated that IL-25, alone or synergized with IL-4, induced an up-regulation of IL-13 in macrophages [18]. It remains to be determined whether macrophages can produce type 2 cytokines during nematode infection or in response to IL-25/IL-33.

In the current study, we demonstrated that macrophages are one of the IL-25/IL-33-responsive cells that play an important role in the induction of type 2 immunity. Macrophages produced various type 2-related cytokines/mediators in response to IL-25/IL-33 stimulation *in vitro* independently of IL-4Rα or STAT6 pathway. Tissues resident macrophages in the spleen, small intestine, and peritoneal cavity were also the IL-13-producing cells in mice receiving exogenously-administrated IL-33 or inoculation of *Heligmosomoides bakeri* (*H. bakeri*). The functional importance of macrophages producing IL-5/IL-13 was established by studies showing that IL-25/IL-33-induced type 2 immunity was significantly attenuated in mice following macrophage depletion. Finally, adoptive transfer of IL-33-activated macrophages into mice with a chronic *H. bakeri* infection promoted worm expulsion associated with a strong type 2 protective immunity.

## Materials and Methods

### Mice

Wild type (WT) BALB/c and C57BL/6 (WT) mice, and mice deficient in STAT6 (STAT6^−/−^), Rag2 (Rag2^−/−^), IL-4Rα (IL-4Rα^−/−^) were purchased from the Small Animal Division of the National Cancer Institute or Jackson Laboratory (Bar Harbor, ME 04609). STAT6^−/−^ or IL-4Rα^−/−^ mice were crossed and back-crossed with Rag2^−/−^ mice to generate STAT6xRag2 or IL-4RαxRag2 double-knockout mice. All animal studies were conducted in accordance with principles set forth in the Guide for Care and Use of Laboratory Animals, Institute of Laboratory Animal Resources, National Research Council, Health and Human Services Publication (National Institutes of Health 85-23, revised 1996), and the Beltsville Animal Care and Use Committee, 2003. All animal studies were approved by the institutional Animal Care and Use Committee.

### Macrophage Preparation, Culture, Enrichment, Adoptive Transfer, and Depletion

Bone marrow mononuclear cells were obtained from mice by flushing the marrow from tibia and femurs into HyClone MEM Alpha medium (Thermo) containing 10% FBS. Cells were cultured overnight at 37°C with 8% CO_2_ in HyClone MEM Alpha medium to deplete adherent stromal cells. The non-adherent mononuclear cells were collected, treated with Red Blood Cell Lysis Buffer to deplete red blood cells. These cells were then cultured in the presence of 20 ng/ml recombinant mouse M-CSF (R & D Systems, Minneapolis, MN) for 7 days with medium changed every other day to generate fully differentiated macrophages. Peritoneal macrophages were prepared as described [19]. MicroBeads (Miltenyi Biotec Inc, Auburn, CA) were further used for macrophages enrichment from PECs per manufacture’s instruction, and the purity of the cells was confirmed by immunofluorescence staining, flow cytometry, or Cytospin. For adoptive transfer, macrophages were prepared as described above, washed, and re-suspended in cold PBS. Cells were adoptively transferred into mice infected with *H bakeri* (1 ml PBS containing ∼1×10^6^ cells per mouse, *i.p.*). For macrophage depletion, 0.2 ml of clodronate-containing liposomes was *i.v.* injected into mice every other day starting 6 days before the cytokine administration. IL-25 or IL-33 (1 µg/mouse), or BSA as control, were *i.p.* injected into mice daily for three days.

### Administration of IL-25 or IL-33

Mice (n = 5/group) were injected *i.v.* or *i.p.* with 1 µg of mouse recombinant IL-25 or IL-33 (R&D Systems, Minneapolis, MN) in 100 µl of saline daily for 3 days or as indicated otherwise. Control mice were given injections of BSA in 100 µl of saline (35 µg/mouse/day). The amount of cytokine administered was from the previous observation that this dose of IL-25 or IL-33 induces a prominent type 2 immune response [1], [3].

### Enteric Nematode Infection and Worm Expulsion

Infective, ensheathed, third stage larvae of *Heligmosomoides bakeri* (*H. bakeri*, L_3_, specimens on file at the U.S. National Parasite Collection, U.S. National Helminthological Collection, Collection 81930, Beltsville, MD) were propagated and stored at 4°C until used [20]. Groups of mice were inoculated orally with 200 *H. bakeri* L_3_. For a secondary inoculation, infected mice were cured with the antihelminthic drug, pyrantel tartrate, 3 weeks after primary inoculation. These mice were reinfected orally with 200 *H. bakeri* L_3_ 10–20 days later. Appropriate age-matched controls were performed for each infection. Determination of adult worm numbers and egg production in feces were performed as described [21].

### In vitro Smooth Muscle Contractility in Organ Baths and Epithelial Cell Ion Transport in Ussing Chambers

*In vitro* smooth muscle contractility was measured as described previously [20]. Smooth muscle responses to electric field stimulation (EFS) or acetylcholine, and the amplitude of spontaneous contractions were determined. Tension was expressed as force per cross sectional area [22]. Muscle-free segments of small intestine were mounted in Ussing chambers as described previously [23]. After a 15-minute period, concentration-dependent changes in short-circuit current (I_sc_) were determined in response to the cumulative addition of acetylcholine to the serosal side. Responses from all tissue segments exposed to acetylcholine from an individual animal were averaged to yield a mean response per animal.

### Micro-snap Well Assay for Mucosal Transepithelial Electrical Resistance (TEER)

The modified micro-snap well system is a miniaturized version of the standard Ussing chamber that has been engineered to measure the TEER of intestinal fragments exposed to various stimuli [24]. A decrease in TEER reflects increased tissue permeability. Briefly, segments of mouse intestine stripped of both muscle and serosal layers were placed in the micro-snap well system. Two hundred fifty µl of DMEM containing 4.5 g/L glucose, 4 mM L-glutamine, 50 U/ml penicillin, 50µg/ml streptomycin, and MEM with 1 mM non-essential amino acids was added to the mucosal side. Three milliliter of the same medium is added to the serosal side. The system was incubated at 37°C with 5%CO_2_ for 30 minutes to stabilize the pH and the baseline TEER measurement was measured.

### RNA Extraction, cDNA Synthesis, Real-time Quantitative Polymerase Chain reaction (qPCR), and ELISA

Total RNA was extracted as described previously [25]. RNA samples (2 µg) were reverse-transcribed to cDNA using the First Strand cDNA Synthase Kit (MBI Fermentas, Hanover, MD) with random hexamer primer. Real-time quantitative PCR was performed on an iCycler detection system (Bio-Rad, CA). PCR was performed in a 25 µl volume using SYBR green Supermix (Bio-Rad, Hercules, CA). Amplification conditions were: 95°C for 3 min, 50 cycles of 95°C for 15 s, 60°C for 15 s, and 72°C for 20 s. The fold-changes in mRNA expressions for targeted genes were relative to the respective vehicle groups of mice after normalization to 18s rRNA. Primer sequences were designed by using Beacon Designer 7.0 (Premier Biosoft International, Palo Alto, CA), and synthesized by the Biopolymer Laboratory of the University of Maryland or Sigma-Aldrich. IL-13 secretion from culture supernatant of macrophages or *in situ* production of IL-13 from tissues isolation was analyzed by ELISA per manufacturer’s instruction (eBioscience, San Diego, CA).

### Immunofluorescence Staining and Flow Cytometry

Frozen blocks of intestine were prepared using the Swiss-roll technique and stored at −80°C. Tissue sections were cut from frozen blocks of intestine or spleen using an HM505E cryostat (Richard-Allan Scientific, Kalamazoo, MI). Tissue slides were fixed in cold acetone for 30 min and blocked with 10% normal donkey serum in PBS for 1 hr at room temperature, incubated with anti-F4/80 (1∶100) and anti-IL-13 (1∶20) antibodies (R&D Systems, Minneapolis, MN) overnight at 4^o^C, and then incubated with DyLight 488 donkey anti-rat IgG and Dylight 649 donkey anti-goat IgG. The slides were cover-slipped with Vectorshield (Vector Laboratories, Burlingame, CA) and digitally photographed using an Olympus microscope and Fluo View confocal software (Olympus America Inc., Centeral Valley, PA). The images were taken by establishing settings for the samples from the individual vehicle groups and using the same conditions to evaluate the samples from the infected or treated groups. Comparisons were made only among slides prepared on the same day. For immunofluorescence staining on macrophages, cells were cultured on Chamber slides and stained as described above. Flow cytometry was carried out to examine the BMDM, PECs, or CD11b MicroBead-enriched macrophages by surface staining with Alexa 488-anti-F4/80 and intracellular staining with PE-anti-IL-13 (eBioscience, San Diego, CA). The cells were analyzed by BD FACScan Flow Cytometer (Franklin Lakes, NJ 07417).

### Solutions and Drugs

Krebs buffer contained (in mM) 4.74 KCl, 2.54 CaCl_2_, 118.5 NaCl, 1.19 NaH_2_PO_4_, 1.19 MgSO_4_, 25.0 NaHCO_3_, and 11.0 glucose. All drugs were obtained from Sigma (St Louis, MO) unless indicated otherwise. On the day of the experiment, acetylcholine was dissolved in water and appropriate dilutions were made.

### Data Analysis

Agonist responses for the *in vitro* smooth muscle contraction were fitted to sigmoid curves (Graphpad, San Diego, CA). Statistical analysis for data presented in bar graphs or tables was performed using one-way ANOVA followed by Neuman-Keuls test. Statistical analysis for comparisons between two curves was performed using two-way ANOVA with repeated measures.

## Results

### Macrophages Produce Type 2 Cytokines/Mediators in Response to IL-25/IL-33 Stimulation *in vitro*

**Figure 1:**
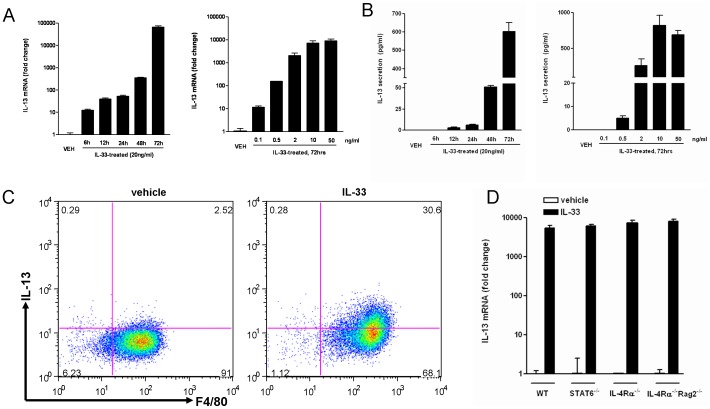
IL-33 stimulated macrophages to produce IL-13 *in vitro*. A, B, Time- and concentration-dependent upregulation of IL-13 mRNA expression (A, qPCR) or IL-13 secretion from cell culture supernatants (B, ELISA) in BMDM stimulated with IL-33. C, After IL-33 stimulation, BMDM were stained with antibodies, and analyzed by FACS for surface F4/80 and intracellular IL-13 expression. D, BMDM from WT, STAT6^−/−^, IL-4Rα^−/−^, or IL-4Rα^−/−^Rag2^−/−^ mice were stimulated with IL-33, and analyzed for IL-13 mRNA expression (72 hours) by qPCR. Data shown in bar graphs are the mean ± s.e.m and representative of at least two independent experiments.

To determine whether macrophages are able to produce type 2 cytokines, we first stimulated bone marrow-derived macrophages (BMDM) with IL-25 or IL-33 directly. BMDM were differentiated from precursors with M-CSF for 7 days and are generally considered to be a pure population of mature macrophages [19]. qPCR showed the expression of IL-25R (IL-17RA and RB) and IL-33R (IL-1RL1 and IL-1Racp) were detected readily in BMDM (not shown), suggesting these cells can respond to IL-25 and IL-33. Indeed, stimulating BMDM with IL-33 dramatically upregulated the IL-13 transcript in a time- and concentration-dependent manner (Fig. 1A). The presence of anti-IL-33 antibody significantly decreased the IL-33 effect on IL-13 expression confirming the specificity of the IL-33 action (18179±847 in IL-33 verses 6427±704 in IL-33 plus anti-IL-33, mRNA in fold changes, IL-33 was at 10 ng/ml for 72 hours, p<0.05, *t* test). ELISA results validated IL-13 protein secretion from the culture supernatants of IL-33-stimulated BMDM (Fig. 1B). FACS analysis showed that ∼95% of the BMDM are F4/80^+^ cells and stimulation with IL-33 significantly increased the cell population that express IL-13 (Fig. 1C).

ILC2 are a potent source of type 2 cytokines in response to stimulation by IL-25 or IL-33. It is, therefore, possible that contamination with ILC2 could expand and contribute to IL-13 production in the BMDM preparation after stimulation with IL-33. To rule this out, we further analyzed the gene expression of the molecular markers for ILC2 using qPCR. Thy1, a marker expressed by all the ILC2, was undetectable in the BMDM before and after IL-33 treatment. In addition, expression of other ILC2 markers, including IL-7R (CD127), c-kit (CD117), and Ly6a (Sca1), was unchanged at all time points examined post IL-33 treatment (data not shown). Moreover, IL-33 treatment of BMDM generated from Rag2/γc double knockout mice, which are devoid of ILCs, induced a significant up-regulation of IL-13 mRNA expression (781±130 in IL-33 versus 1.0±0.1 in vehicle, fold change). Overall, these data demonstrated that ILC2 were not present at significant number nor expanded by IL-33 treatment in our BMDM preparation supporting the conclusion that IL-13 was produced by macrophages rather than ILC2.

IL-33 stimulation of BMDM significantly upregulated the expression of other type 2-related mediators/chemokines such as IL-5, IL-9, GATA-3, Junb, Maf, CCL2, CCL17, and CCL24 (Table 1). The cell lineage/maturation markers, CD11b and CD86, and the IL-33 receptor subunit ST2, were upregulated modestly by IL-33. The expression of IL-4 was extremely low even after stimulation. IL-33 did not affect the mRNA expression of T-regulatory cytokines IL-10 and TGFβ1, but downregulated several Th1/Th17-related genes including T-bet, IL-12p40, IL-17RA, implying it may have anti-inflammatory activities (Table 1).

**Table 1:** Genes regulated by IL-33 in BMDM (mRNA expression by real-time qPCR).

	Genes	VEH	IL-33 (20 ng/ml) 72 hours
Up-regulated	*IL13*	1±0.1	43238±4864
	*IL4*	1±0.1	4.8±0.5
	*IL5*	1±0.2	1552±396
	*ARG1*	1±0.1	416±62
	*CD206*	1±0.1	2.2±0.2
	*YM-1*	1±0.1	15.5±7.7
	*NOS2*	1±0.2	7.6±0.2
	*IFNγ*	1±0.4	5.7±1.2
	*IL6*	1±0	15.6±1
	*GATA3*	1±0.1	5.7±1
	*Junb*	1±0.1	2.3±0.4 (24 hour)
	*Maf*	1±0.5	3.4±0.7
	*CCL2*	1±0.1	9.8±0.6
	*CCL17*	1±0.1	5±1
	*CCL24*	1±0	9.6±1.7
	*CD11b*	1±0.1	1.5±0.1
	*CD86*	1±0	1.5±0
	*IL1RL1*	1±0	2.2±0.4
Down-regulated	*DEC2*	1±0.1	0.5±0
	*IL17RA*	1±0	0.5±0
	*Tbx21*	1±0.1	0.3±0.1
	*TGFβ_3_*	1±0	0.4±0
	*IL12p40*	1±0.4	0.1±0

BMDM: bone marrow-derived macrophages.

STAT6 is a master transcription factor important for type 2 immunity and is involved in host defense against nematode infection and development of allergic inflammation [26]. To determine if STAT6 was required for the IL-33 effects, we generated BMDM from STAT6^−/−^ mice. Comparable upregulation of IL-13 mRNA was observed in WT and STAT6^−/−^ cells following IL-33 stimulation *in vitro,* indicating the independence of STAT6 (Fig. 1D). In addition, the IL-33-induced up-regulation of IL-13 remained in the BMDM generated from STAT6^−/−/^Rag2^−/−^ or IL-4Rα^−/−/^Rag2^−/−^ mice that lack T and B cells (Fig. 1D and not shown), confirming that IL-13 was not from any contaminating lymphocytes.

Similar results were obtained from IL-25-stimulated BMDM in that various type 2-related cytokines/mediator were upregulated (Fig. S1). Interestingly, IL-25 shared many activities on macrophages with IL-33, but no additive or synergetic effects were observed (data not shown), suggesting they may share some common downstream signaling pathways for their biological activities. It is noteworthy that IL-33 is a more potent type 2 cytokine inducer in a BMDM preparation than IL-25, as a relatively lower concentration of IL-33 is needed for the full extent of activation.

### IL-33 Induces Alternative Activation of Macrophages

**Figure 2:**
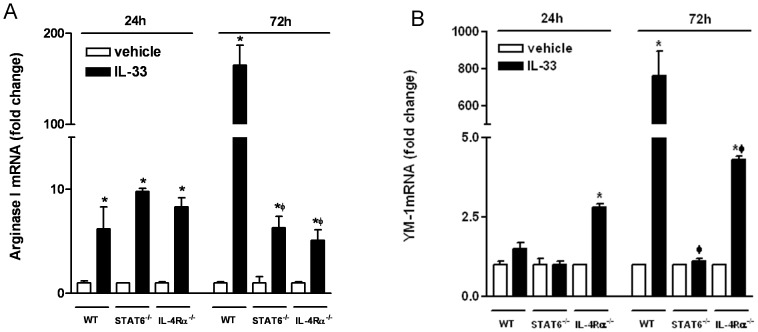
IL-33 induced alternative activation of macrophages *in vitro*. BMDM from WT, STAT6^−/−^, or IL-4Rα^−/−^ mice were stimulated with IL-33 (24 and 72 hours), and analyzed for the expression of arginase I (A) or YM-1 (B) by qPCR (*p<0.01 vs respective vehicle; ^φ^p<0.001 vs WT-IL-33, one-way ANOVA followed by Neuman-Keuls test). Data shown in bar graphs are the mean ± s.e.m and representative of two independent experiments with similar results.

Depending on the cytokine microenvironment, macrophages undergo distinct pathways of activation: the classically-activated macrophages (M1) induced by the Th1 cytokine IFN-γ plus LPS, or the alternatively-activated macrophages (M2) induced by Th2 cytokines, IL-4 and IL-13 [27]. The ability of IL-33 to induce IL-13 production from macrophages implies that this cytokine may be capable of alternatively activating macrophages through an autocrine pathway. Indeed, most of the M2 markers were upregulated significantly in BMDM after IL-33 stimulation including arginase I, CD206, and YM-1 (Fig. 2 and Table). To further dissect the underlying mechanism, we used BMDM from STAT6^−/−^ and IL-4Rα^−/−^ mice. Interestingly, IL-33 induced a modest, but significant, upregulation of arginase I at an early time point (24 hours), which was comparable among WT, STAT6^−/−^, and IL-4Rα^−/−^ cells (Fig. 2A). At a later time point (72 hours), however, the upregulation of arginase I by IL-33 was highly elevated in WT BMDM but was significantly less in STAT6^−/−^ or IL-4Rα^−/−^ cells (Fig. 2A). Similar results were observed in the expression of YM-1, another M2 marker (Fig. 2B). These data indicated that IL-33 can alternatively activate macrophages primarily through an autocrine IL-13-IL-4Rα-STAT6-dependent pathway.

### Macrophages are IL-13-producing Cells *in vivo*

**Figure 3:**
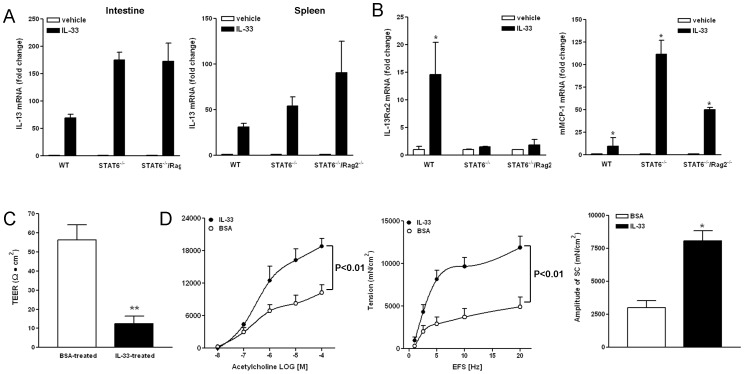
Exogenously administrated IL-33 in mice promoted type 2 immunity. Mice were *i.p.* injected with BSA or IL-33 (1 µg/mouse) daily for 3 days. Tissues were collected for functional or molecular analysis. A, IL-33 injection increased IL-13 expression in the small intestine and spleen independently of STAT6 or T/B cells. B, mRNA expression of IL-13Rα2 or mast cell protease I (mMCP-1) in the small intestine (n = 3–5 mice per group, *p<0.05 vs respective vehicle for A&B). C, IL-33 injection induced increase in the permeability of intestinal mucosa (decrease in transepithelial electrical resistance, TEER) (n = 5 per group, **p<0.01 vs BSA-treated, *t* test). D, IL-33 induced intestinal smooth muscle hypercontractility. Smooth muscle responses to acetylcholine and electric field stimulation (EFS), as well as amplitude of spontaneous contraction (SC) are shown (n = 5 per group, p<0.01 was generated from two-way ANOVA with repeated measures between the two curves). Data shown in bar and line graphs are the mean ± s.e.m and representative of two independent experiments with similar results.

To determine if the *in vitro* activity of IL-25/33-stimulated macrophages to produce type 2 cytokines could be replicated *in vivo*, we injected mice with IL-25 or IL-33 daily for three days and collected small intestine and spleen for molecular analysis and/or functional studies. As expected, IL-13 was upregulated in both tissues examined (Fig. 3A). In addition, upregulation of IL-13 was observed in the respective tissues from STAT6^−/−^ mice indicating that the *in vivo* type 2 cytokine response induced by IL-33 was independent of STAT6 (Fig. 3A). Adaptive immunity is important for host protective immunity against enteric nematode infection, however, IL-33-induced upregulation of IL-13 in STAT6^−/−/^Rag2^−/−^ mice was no less than that of WT mice (Fig. 3A), indicating that T/B cell-mediated adaptive immune response was not required for this activity of IL-33 *in vivo*. IL-33 injection also induced a comparable upregulation of IL-5 in the intestine and spleen from WT, STAT6^−/−^, as well as STAT6^−/−/^Rag2^−/−^ mice (data not shown). It is known that IL-13 activation of STAT6 during nematode infection increases the expression of IL-13Rα2, 5-HT_2A_, and arginase I in WT [13], [25], [28]. Not surprisingly, exogenous IL-33 upregulated the expression of these genes in WT, but not STAT6^−/−^ or STAT6^−/−/^Rag2^−/−^ intestine (Fig. 3B and data not shown). On the other hand, a modest upregulation of mMCP-1, a marker for murine mast cells, was observed in IL-33-treated WT intestine and this upregulation was even more dramatic in STAT6^−/−^ or STAT6^−/−/^Rag2^−/−^ intestine, suggesting a STAT6/Rag2-independent effect of IL-33 (Fig. 3B). The characteristic changes in intestinal function, including the increase in mucosal permeability (decrease in trans-epithelial electrical resistance, TEER, Fig. 3C), smooth muscle hypercontractility (Fig. 3D), and epithelial hyposecretion (not shown), were also detected in mice injected with IL-33. These changes were similar to those reported previously in response to nematode infection or exogenous administration of IL-4/IL-13 or IL-25 [1], [20], [23], [28]–[30].

**Figure 4:**
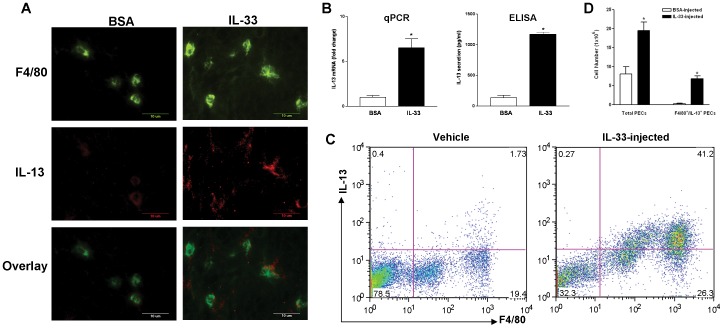
Exogenously administrated IL-33 in mice induced IL-13-expressing macrophages. C57BL/6 mice were *i.p.* injected with BSA or IL-33 (1 µg/mouse) daily for 3 days. A, Sections of small intestine were stained with anti-F4/80 (green) and anti-IL-13 (red). Representative images from 5 animals per group were shown. B, Peritoneal exudate cells (PECs) were collected and analyzed for IL-13 expression by qPCR or ELISA in triplicates (n = 3–5 per group, *p<0.05 vs respective BSA, *t* test). C, Representative plots from PECs stained with anti-F4/80 and anti-IL-13 for analyzing IL-13-expressing macrophages by FACS. D, Number of total PECs and F4/80^+^/IL-13^+^ PECs collected per mouse after injection of BSA or IL-33 (n = 3–5 per group, *p<0.05 vs respective BSA). Data shown in bar graphs are the mean ± s.e.m and representative of at least two independent experiments with similar results.

To locate the *in situ* IL-13 expressing cells, sections of intestine were double-stained with anti-F4/80 and anti-IL-13. In control mice, only weak staining of IL-13 was visualized in F4/80^+^ macrophages in the lamina propria of small intestine. In IL-33-treated mice, a significantly stronger staining of IL-13 was detected inside and around the F4/80^+^ macrophages (Fig. 4A). The number of IL-13-expressing F4/80^+^ macrophages was increased also in jejunal sections from mice that were given a secondary inoculation of *H. bakeri* to elicit potent type 2 memory response (Fig. S2). These data indicate that macrophage expression of IL-13 is a common feature of the type 2 protective immunity.

Peritoneal exudate cells (PECs) were collected by peritoneal wash with PBS for subsequent analysis of IL-13 expression. The PECs from IL-33-treated mice expressed significantly higher levels of IL-13 mRNA (qPCR) compared with those from vehicle-treated mice (Fig. 4B). Correspondingly, more IL-13 was produced in the peritoneal lavage of IL-33-treated mice (Fig. 4B). Likewise, the IL-33-induced upregulation of IL-13 in PECs did not require STAT6 pathway nor the T/B cell-mediated adaptive immune response, as the cells from STAT6^−/−/^Rag2^−/−^ mice had an even higher level of IL-13 transcript than that from WT mice after injection with IL-33 (WT, 6.5±1.0 vs STAT6^−/−/^Rag2^−/−^,109±25). Most of the PECs from IL-33-treated mice were adherent after they were plated and cultured for ∼2 hours consistent with their identification as macrophages. FACS analysis of PECs showed that IL-33 treatment dramatically increased the population of F4/80^+^ macrophages (∼70%) and the IL-13-expressing macrophages (∼40%) (Fig. 4C). Furthermore, when compared with BSA-injected mice, administration of IL-33 increased the number of total PECs isolated, as well as the number and ratio of macrophages expressing IL-13 (F4/80^+^/IL-13^+^) (Fig. 4D).

### Macrophage Depletion *in vivo* Impairs IL-25/IL-33-induced Type 2 Immunity

**Figure 5:**
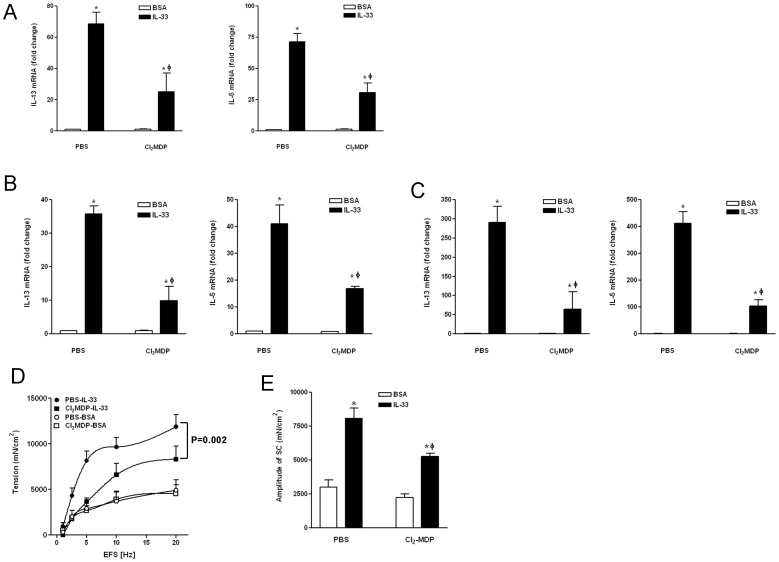
Macrophage depletion in mice impaired type 2 immunity induced by exogenous IL-33. A–C, IL-33-induced upregulation of IL-13 and IL-5 was attenuated in the small intestine (A), spleen (B), and mesenteric lymph nodes (C), in mice treated with Cl_2_MDP. D, E, IL-33-induced smooth muscle hypercontractility was attenuated in mice with decreased number of macrophage as shown by the response to electric field stimulation (D, EFS) and the amplitude of spontaneous contraction (E, SC) (n = 5 per group, *p<0.05 vs respective BSA; ^φ^p<0.05 vs PBS-IL-33, one-way ANOVA followed by Neuman-Keuls test; p = 0.002 was generated from two-way ANOVA with repeated measures between the two curves). Data shown in bar and line graphs are the means ± s.e.m, and are representative of two independent experiments.

After establishing that macrophages were capable of responding to IL-25/IL-33 and producing type 2 cytokines/mediators, we sought to investigate if macrophages were essential for the *in vivo* induction of type 2 immunity in response to IL-25/IL-33. Groups of mice were treated with clodronate-containing liposomes (Cl_2_MDP) to deplete macrophages or PBS-liposomes as controls, and then injected with IL-33 or BSA (vehicle). Cl_2_MDP treatment decreased the number of tissue macrophages, as shown by the significantly decreased expression of F4/80 in the small intestine (∼50%) and spleen (∼90%). Correspondingly, the IL-33-induced IL-5 and IL-13 cytokine response in small intestine (Fig. 5A), spleen (Fig. 5B), and mesenteric lymph nodes (Fig. 5C) was significantly attenuated in mice treated with Cl_2_MDP compared to PBS-liposomes-treated controls. In addition, the IL-33-induced intestinal smooth muscle hyper-contractility to electric field stimulation (Fig. 5D) and increase in the amplitude of spontaneous contraction (Fig. 5E) were significantly attenuated in mice with decreased number of macrophages. We observed similar results in mice treated with IL-25 (Fig. S3). Overall, these data indicate that macrophages are essential for a full development of type 2 immunity induced by IL-33 or IL-25.

### Adoptive Transfer of IL-33-activated Macrophages into Mice Confers a Protective Immunity against Chronic Nematode Infection

Both IL-25 and IL-33 are upregulated during nematode infection and play an important role in host protective immunity [1], [2], [31]. In the case of *Nippostrongylus brasiliensis* infection, macrophage accumulation starts at day 4 post infection (PI) and precedes the peak IL-4/13 expression at day 7 PI and maximal gut functional alterations at day 9 PI [28]. Monitoring the sequential changes in the expression of cytokines following the infection showed that IL-33 expression peaked at day 6 PI, followed by the peak IL-4/IL-13 expression [28], suggesting that IL-33 plays a role in the initiation/amplification of the type 2-mediated immune response.

**Figure 6:**
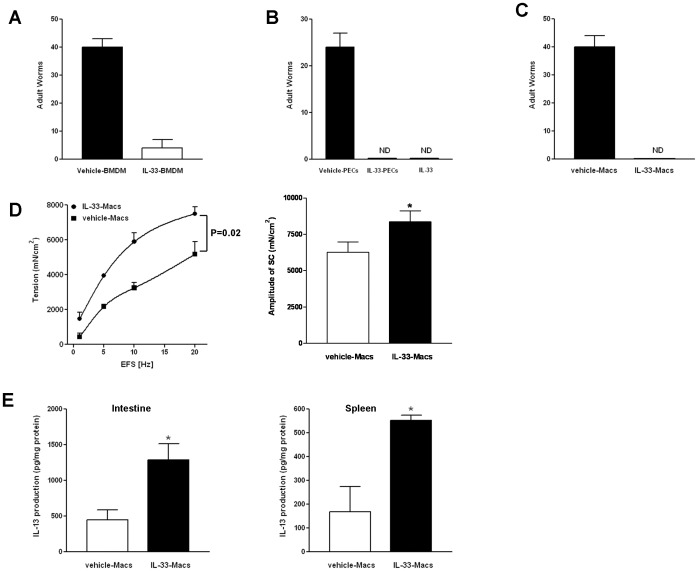
Adoptive transfer of IL-33-activated macrophages promoted protective immunity against *H. bakeri (Hb)* infection. A–C, Numbers of adult worms in the lumen of small intestine in mice receiving BMDM (A), PECs (B), or CD11b MicroBead-enriched PECs (C). D, E, Enhanced type 2 immunity in mice that received IL-33-activated CD11b MicroBead-enriched PECs (IL-33-Macs). Intestinal smooth muscle function (D) and *in situ* IL-13 production in the small intestine and spleen (E) were shown. Data shown in bar and line graphs are the mean ± s.e.m, and are representative of two independent experiments (n = 3–6 mice per group, *p<0.05 vs respective vehicle-Macs, *t* test; p = 0.02 was generated from two-way ANOVA with repeated measures between the two curves). ND, not detected.

To further address the functional importance of macrophages in mediating the IL-25/IL-33 effects on type 2 immunity, we performed adoptive transfer of IL-33-activated macrophages to mice after primary inoculation with *H. bakeri* which would normally develop chronic infection with persistent adult worms in the intestinal lumen for months due to insufficient type 2 immunity. Fecal worm egg counts (worm fecundity) were performed before transfer to establish that the infections were similar between treatment groups. Worm fecundity was monitored continuously following the transfer and the number of adult worms in the lumen was counted at the time of euthanasia as a primary index of host immunity. In the first set of experiments, we transferred BMDM that were stimulated with IL-33 *in vitro* for 72 hours. Mice receiving IL-33-activated BMDM had a significantly reduced number of fecal worm eggs when compared to mice transferred with vehicle-treated BMDM (data not shown). More importantly, all of the mice receiving vehicle-BMDM had significant numbers of adult worms while five out of six mice that received IL-33-BMDM had no worms at euthanasia (Fig. 6A). A second set of experiments was carried out by adoptively transferring PECs collected from IL-33- or BSA-injected mice. Likewise, mice receiving IL-33-PECs had fewer numbers of fecal worm eggs following the transfer, and these mice expelled all worms while the control group of mice given BSA-treated PECs still harbored a significant number of worms at euthanasia (Fig. 6B and not shown). The protective effect of adoptive transfer of IL-33-treated PECs on host immunity was comparable to that of exogenous IL-33 injections (Fig. 6B). The isolated PECs contained ∼30% non-F4/80^+^ cells as shown by flow cytometry. To ensure that the effect of IL-33-treated PECs was due to macrophages rather than other cell populations, we further enriched macrophages from PECs using CD11b Microbeads. Enrichment increased the IL-13-expressing cell population, as shown by the significantly higher IL-13 mRNA expression of Microbead-bound cell (CD11b^+^) than that of unbound cells (Fig. S4A). In addition, the majority of these enriched cells were adherent when they were plated and cultured for ∼2 hours confirming the macrophage nature of these cells. FACS analysis and immunofluorescence staining also validated the purity (>90% F4/80^+^) and IL-13-producing properties of these enriched macrophages (Fig. S4B, S4C). Finally, adoptive transfer of these cells into *H. bakeri*-infected mice induced protective immunity (Fig. 6C). Worm clearance induced by adoptive transfer of CD11b MicroBead-enriched IL-33-treated PECs was associated with an enhanced intestinal smooth muscle hyper-contractility (Fig. 6D) and a significantly increased *in situ* IL-13 production in the spleen and intestine (Fig. 6E), which in turn lead to decreased worm fecundity and accelerated worm expulsion.

## Discussion

It is well established that enteric nematode infections induce a polarized type 2 cytokine response that is essential to the host protective immunity, however, there is little information on the effector cells and molecules involved in the initiation, maintenance, and resolution of the immune cascade [32]. IL-25 and IL-33 are predominately epithelium-derived cytokines implicated in the induction of type 2 immunity [1], [33], [34]. In the present study, we provide evidence that macrophages are one of the IL-25/IL-33 responsive cells that can produce type 2 cytokines and mediate protective immunity against intestinal nematodes.

IL-25 and IL-33 have recognized roles in the promotion of type 2 immunity but identification of the responsive cells, particularly those involved in the initiation of type 2 response, has not been fully elucidated. IL-33 is a recently identified member of the IL-1 cytokine family that is widely expressed by many cell types including major innate immune cells (macrophages and dendritic cells), Th2 cells, B1 cells, and non-hematopoietic structural cells particularly epithelial and endothelial cells [34], [35]. IL-25 is a member of the IL-17 cytokine family with highest level in the gastrointestinal tract and lung, where it is expressed primarily by epithelial cells and perhaps also on immune cells [1], [3], [36]. IL-33 binds to a heterodimer receptor composed of ST2 and IL-1R accessory protein, leading to activation of NF-κB and MAPKs pathways. Previous studies show that IL-33 treatment can differentiate naïve T cells into Th2 cells or amplify Th2 cytokine production from already differentiated Th2 cells [35]. Likewise, *in vitro* IL-33 can stimulate mast cells or basophils to produce type 2-related cytokine/chemokines such as IL-5 and IL-13 [34]. IL-25R is also a heterodimer consisting of IL-17RB and IL-17RA, a receptor subunit shared by other proinflammatory cytokine members of the IL-17 family. An early study suggests a population of non-T/non-B cells can respond to IL-25. Until recently, novel cell populations, namely “natural helper cells”, “nuocytes”, and “innate helper type 2 cells”, were identified as IL-25/IL-33-responsive cells [4]–[7]. These ILC2 cells share several common features: lacking known cell lineage markers, restricted tissue/organ distribution, and low abundance. Importantly, most of these cells do not produce type 2 cytokines in response to direct stimulation of IL-25 or IL-33.

Macrophages are the most abundant innate immune cells distributed throughout the body and are implicated in various types of infection/inflammation. IL-25 was shown to be capable of inhibiting LPS-stimulated production of proinflammatory cytokines from macrophages [37], whereas IL-33 appeared to have synergetic effects on LPS-induced cytokine production and these effects may be mediated by an IL-33-induced increase in the LPS receptor components and MyD88 adaptor molecule [38]. In the current study, we show that macrophages can respond to either IL-25 or IL-33 directly to produce type 2 cytokines, IL-13 and IL-5, and various type 2-related chemokines. The results are consistent with a recent study demonstrating that IL-25, alone or synergized with IL-4, up-regulated the IL-13 expression in BMDM [18]. We were also able to validate the IL-13-producing capacity of macrophages *in vivo* after mice received exogenous IL-33 or enteric nematode infection, where the macrophages in lamina propria of intestine and peritoneal cavity were the major IL-13-expressing cells.

Activation of macrophage results in either M1 or M2 phenotype depending on the cytokine microenvironment. Both IL-25/IL-33 were shown to promote alternative activation of macrophages *in vivo* possibly via the downstream IL-4 and IL-13 [34], [39]. Using human bone marrow-derived macrophages, Joshi et al. showed that IL-33 induced M1 development from naïve cells, but promoted the expression of M2-associated chemokines from activated macrophages [40]. We showed here that IL-33 can alternatively activate macrophages *in vitro* as evidenced by the up-regulation of M2 markers such as arginase I, CD206, and YM-1. We further established that the IL-33-induced activation of macrophages is primarily through autocrine IL-13 activating IL-4Rα-STAT6 pathway.

Exogenous IL-33 induced up-regulation of IL-5 and IL-13 consistently with its role in promoting type 2 immunity. The independence of the IL-33 effect on STAT6 pathway is in agreement with the previous study in that STAT6^−/−^ mice have a normal upregulation of type 2 cytokines during *N. brasiliensis* infection despite impaired worm expulsion [41]. T/B cell-mediated adaptive immunity is important for host protective immunity against enteric nematode infection; yet IL-4/IL-13 from innate immune cells are sufficient to induce worm expulsion [42]. We showed that exogenous IL-33 induced a comparable up-regulation of IL-5/IL-13 between WT and Rag2^−/−^ mice confirming that these type 2 cytokines are from innate immune cells, possibly macrophages. Characteristic alterations in intestinal function such as smooth muscle hypercontractility, epithelial hyposecretion, and increased mucosal permeability, are the hallmarks of enteric nematode infection that contribute, at least in part, to worm expulsion [20]. These changes were also observed in mice receiving IL-25 [20] as well as IL-33, implying that both IL-25 and IL-33 may contribute to the host protective immunity against enteric nematode infection via regulating intestinal function.

Several types of innate immune cells are capable of producing IL-4/IL-13 including eosinophils, basophils, and mast cells. For instance, eosinophilia is a consequence of the type 2-mediated inflammation and eosinophil expressing IL-4 is an early event during nematode infection. Mice with eosinophil deficiency, however, have no obvious defect in host protective immunity against the infection [42]. Basophils producing IL-4 and interacting with CD4^+^ T cells were thought to be necessary and sufficient for Th2 induction [43]. This concept has been challenged repeatedly and a very recent study confirmed that basophils were not critical for initiating allergic inflammation or host immunity against nematode infection [44]. Similarly, mast cells can produce IL-5 and IL-13 under certain conditions, whereas W/Wv mice deficient in mast cells expel *N. brasiliensis* or develop allergic inflammation normally. The four lineage-negative novel cell populations are new additions to the list of type 2 cytokine-expressing innate immune cells. Although sufficient to induce type 2 immunity when adoptively transferred to mice, there is no evidence that these cells are required for the onset or maintenance of type 2 immunity.

We and others showed previously that alternatively activated macrophages are necessary for the host protective immunity against enteric nematode infection [12], [13]. In the present study, we further established that macrophages are required for a full development of type 2 immunity *in vivo*, as mice with decreased number of macrophages (treated with clodronate) had an attenuated response to exogenous IL-25 or IL-33. It is important to note that clodronate treatment depletes circulating monocytes, thus preventing only tissue macrophages from being replenished. The incomplete depletion of macrophages by clondronate treatment likely accounts for the residual immune response to exogenous IL-25/IL-33 in these mice. To corroborate the functional importance of macrophage producing type 2 cytokines/mediators, adoptive transfer of IL-33-activated cells into mice chronically infected with *H. bakeri* conferred a strong protective immunity, evidenced by the increased *in situ* type 2 cytokine production, enhanced intestinal smooth muscle hypercontractility, decreased worm fecundity, and accelerated worm expulsion. Thus, macrophages represent a unique population of innate immune cells essential to host defense against nematode infection.

Taken together, these results indicated that macrophages can respond to IL-25/IL-33 and contribute to the induction of type 2 immunity. Coupled with their well-established roles in linking innate to adaptive immunity and in various types of infection/inflammation, macrophages represent a potential therapeutic target in controlling not only Th1- but also type 2-mediated pathologies.

## Supporting Information

Figure S1 **IL-25 stimulated macrophages to produce IL-13** ***in vitro*** **.** A, B, Time- and concentration-dependent upregulation of IL-13 mRNA expression (A, qPCR) or IL-13 secretion from cell culture supernatants (B, ELISA) in BMDM stimulated with IL-25. C, BMDM from WT, STAT6^−/−^, or STAT6^−/−^Rag2^−/−^ mice were stimulated with IL-25 (50 ng/ml) for 48 hours and analyzed for IL-13 mRNA expression by qPCR. D. Anti-IL-25 inhibited the IL-25-induced upregulation of IL-13 in BMDM. Data shown in bar graphs are the mean ± s.e.m and are representative of two independent experiments with similar results.
(TIF)

Figure S2 **IL-13-expressing macrophages in the spleen and intestinal mucosa.** Mice were infected with *Heligmosomoides bakeri* (*H. bakeri)*. Sections of intestine were stained with anti-F4/80 (green) and anti-IL-13 (red). Images are the representative from two independent experiments (n = 5 per group).
(TIF)

Figure S3 **Macrophage depletion** ***in vivo*** **impaird the Th2 immunity induced by exogenous IL-25.** A, B. IL-25-induced upregulation of IL-13 (A) and IL-5 (B) was attenuated in the small intestine and spleen in mice treated with Cl_2_MDP. C-D, IL-25-induced smooth muscle hyper-contractility was attenuated in mice with decreased number of macrophage as shown by the response to electric field stimulation (C, EFS) and the amplitude of spontaneous contraction (D, SC) (n = 5 per group, *p<0.05 vs respective BSA; φp<0.05 vs PBS-IL-33, one-way ANOVA followed by Neuman-Keuls test; p<0.05 was generated from two-way ANOVA with repeated measures between the two curves). Data shown in bar and line graphs are the means ± s.e.m, and are representative of two independent experiments with similar results
(TIF)

Figure S4 **Increased IL-13 expression in CD11b MicroBead-enriched peritoneal exudate cells (PECs) from mice injected with IL-33.** PECs were collected from mice receiving daily injection of IL-33 or BSA for 3 days and were further enriched using CD11b Microbeads. The enriched cells were analyzed for IL-13 mRNA expression by qPCR (A) or surface F4/80 and intracellular IL-13 expression by FACS (B). A separate fraction of the enriched cells were plated in Chamber slides, cultured at 37C for 2 hours, and then stained with DAPI (green), anti-F4/80 (green), and anti-IL-13 (red). The images were visualized under fluorescence microscope. Data shown are representative of two independent experiments (n = 3–5 mice per group).
(TIF)
