# Supplementary figures and images for: Macrophages as IL-25/IL-33-Responsive Cells Play an Important Role in the Induction of Type 2 Immunity
Source: PLoS One. 2013 Mar 25;8(3):e59441. doi: 10.1371/journal.pone.0059441 (PMC3607614; doi:10.1371/journal.pone.0059441)

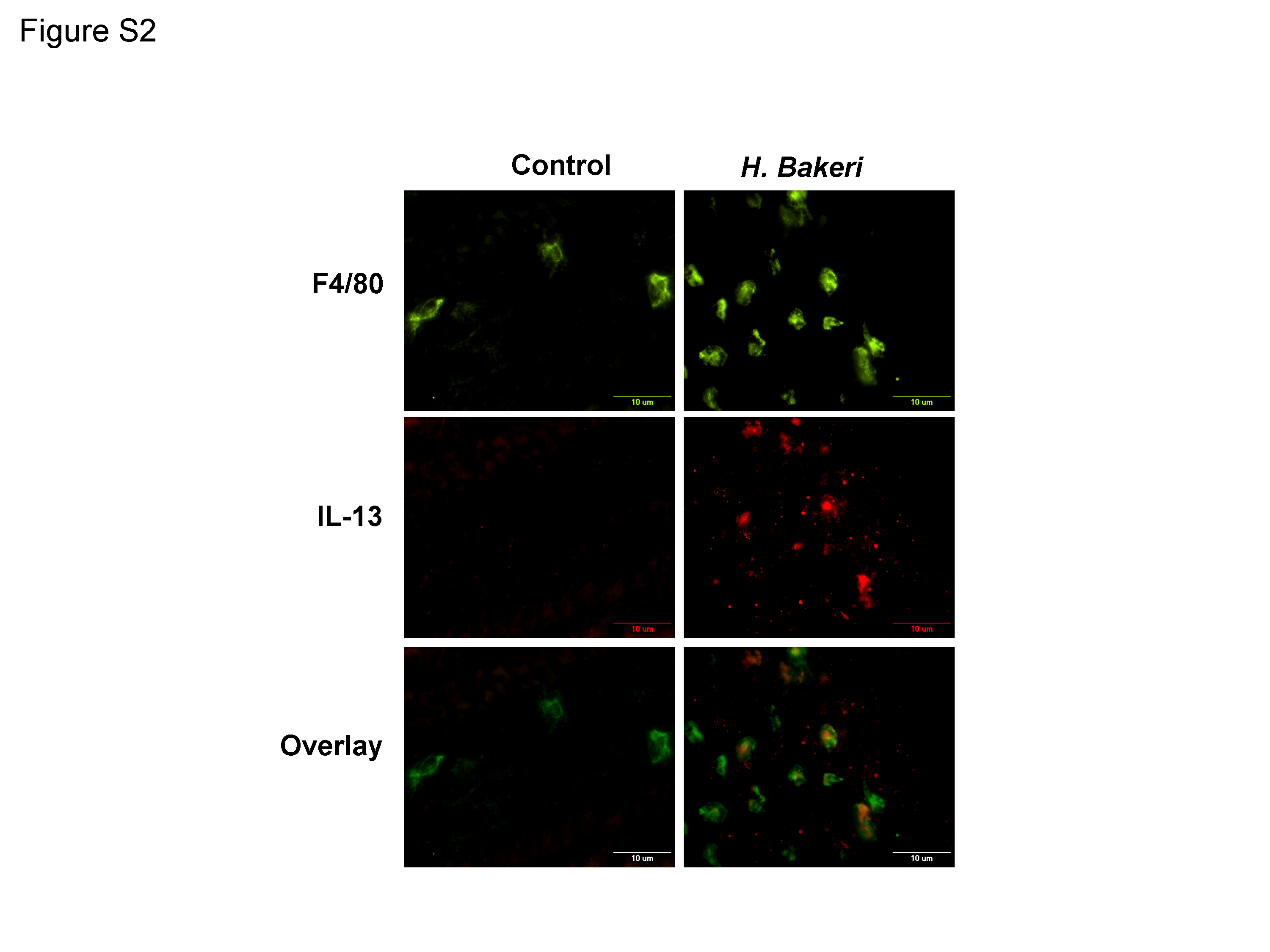

Supplement: Figure S2 — IL-13-expressing macrophages in the spleen and intestinal mucosa. Mice were infected with Heligmosomoides bakeri (H. bakeri). Sections of intestine were stained with anti-F4/80 (green) and anti-IL-13 (red). Images are the representative from two independent experiments (n = 5 per group). (TIF) [file pone.0059441.s002.tif]

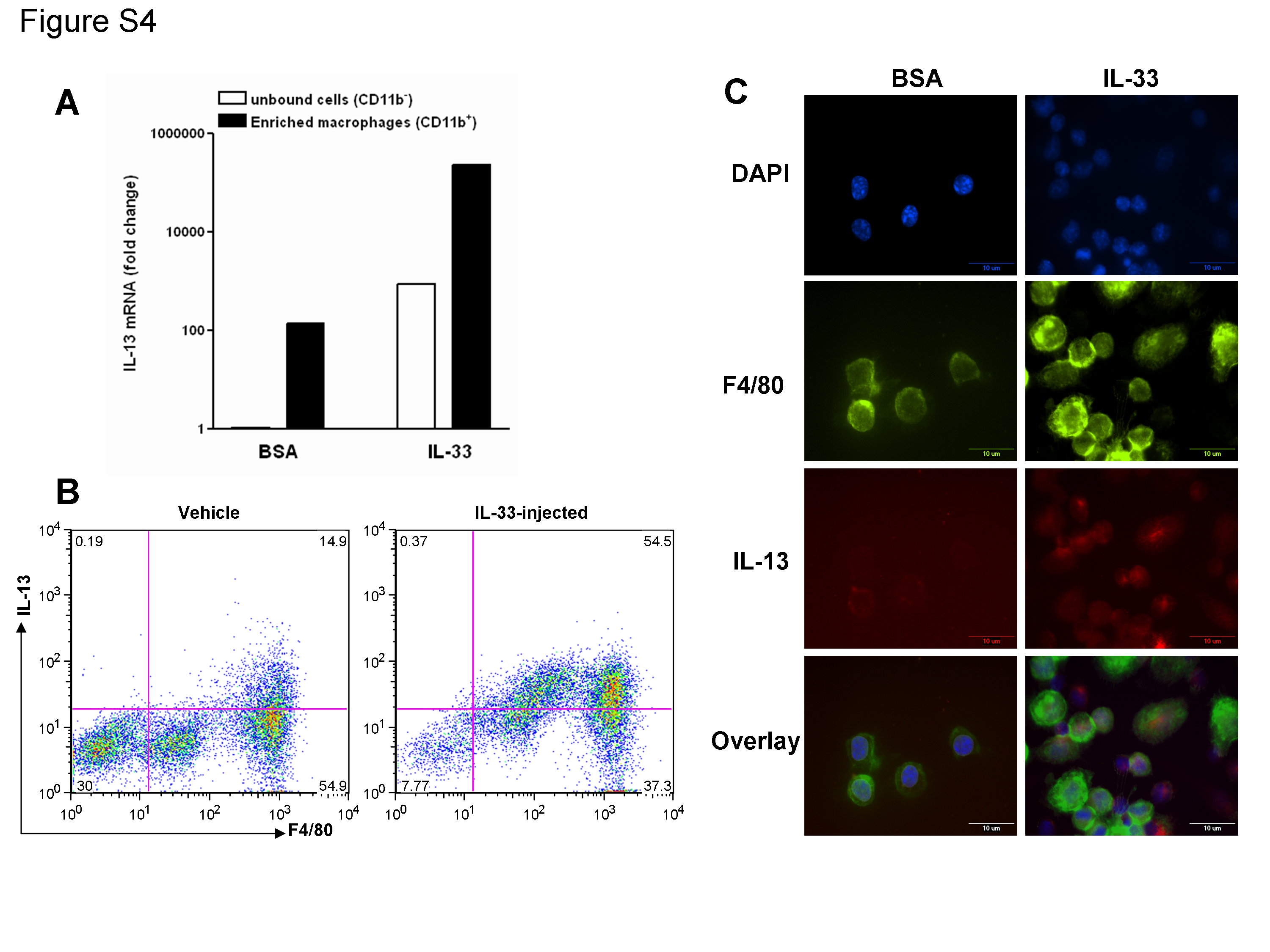

Supplement: Figure S4 — Increased IL-13 expression in CD11b MicroBead-enriched peritoneal exudate cells (PECs) from mice injected with IL-33. PECs were collected from mice receiving daily injection of IL-33 or BSA for 3 days and were further enriched using CD11b Microbeads. The enriched cells were analyzed for IL-13 mRNA expression by qPCR (A) or surface F4/80 and intracellular IL-13 expression by FACS (B). A separate fraction of the enriched cells were plated in Chamber slides, cultured at 37C for 2 hours, and then stained with DAPI (green), anti-F4/80 (green), and anti-IL-13 (red). The images were visualized under fluorescence microscope. Data shown are representative of two independent experiments (n = 3–5 mice per group). (TIF) [file pone.0059441.s004.tif]
